# Transcranial magnetic stimulation treatment in Alzheimer’s disease: a meta-analysis of its efficacy as a function of protocol characteristics and degree of personalization

**DOI:** 10.1007/s00415-022-11236-2

**Published:** 2022-07-04

**Authors:** Arianna Menardi, Lisa Dotti, Ettore Ambrosini, Antonino Vallesi

**Affiliations:** 1 Department of Neuroscience, University of Padova, 35121 Padua, Italy; 2 Padova Neuroscience Center, University of Padova, Padua, Italy; 3 Department of General Psychology, University of Padova, Padua, Italy

**Keywords:** Alzheimer’s disease, Transcranial magnetic stimulation, Personalized medicine, Intervention efficacy, Individualized stimulation

## Abstract

Alzheimer’s disease (AD) represents the most common type of neurodegenerative disorder. Although our knowledge on the causes of AD remains limited and no curative treatments are available, several interventions have been proposed in trying to improve patients’ symptomatology. Among those, transcranial magnetic stimulation (TMS) has been shown a promising, safe and noninvasive intervention to improve global cognitive functioning. Nevertheless, we currently lack agreement between research studies on the optimal stimulation protocol yielding the highest efficacy in these patients. To answer this query, we conducted a systematic literature search in PubMed, PsycINFO and Scopus databases and meta-analysis of studies published in the last 10 years (2010–2021) according to the Preferred Reporting Items for Systematic Reviews and Meta-Analyses (PRISMA) guidelines. Differently from prior published meta-analytic work, we investigated whether protocols that considered participants-specific neuroimaging scans for the selection of *individualized* stimulation targets held more successful outcomes compared to those relying on a *generalized* targeting selection criteria. We then compared the effect sizes of subsets of studies based on additional protocol characteristics (frequency, duration of intervention, number of stimulation sites, use of concomitant cognitive training and patients’ educational level). Our results confirm TMS efficacy in improving global cognitive functioning in mild-to-moderate AD patients, but also highlight the flaws of current protocols characteristics, including a possible lack of sufficient personalization in stimulation protocols.

**Supplementary Information:**

The online version contains supplementary material available at 10.1007/s00415-022-11236-2.

## Introduction

Alzheimer’s disease (AD) represents the most common type of neurodegenerative disease [1]. Due to its multifactorial nature, the gradual cognitive decline is associated with the interaction of multiple pathological alterations, including brain atrophy, amyloid plaques deposition, and neurofibrillary tangles formation, which ultimately lead to a clinical diagnosis of dementia [2–6]. Despite abnormal amyloid markers being recognized as an early indicator or risk factor of AD, it has been suggested to refrain from giving an AD diagnosis to patients with abnormal protein builds-up but normal cognitive functioning [7]. Indeed, the National Institute on Aging and Alzheimer's Association (NIA-AA) recognizes AD as a spectrum characterized by widespread cognitive deficits that extend beyond the well-known memory decline [8]. Yet, little is known about the causes of Alzheimer’s disease and no curative treatments are available [9]. In this complex framework, some unmodifiable risk factors are acknowledged, such as age, family history, cardiovascular pathologies and genetic factors, such as the presence of the Apolipoprotein E4 (APOE4) allele [10–13]. On the other hand, modifiable risk factors are represented by poor diet choices [14, 15], lack of physical exercise [16, 17] and cognitive stimulation [18], as well as hearing loss and the exposure to environmental stress [19], among others. From this perspective, nonpharmacological treatments may play an important role, especially in the form of preventive medicine via general lifestyle choices, as well as the effective management of overall health conditions and cognitive wellness [20].

As a form of nonpharmacological intervention, noninvasive brain stimulation techniques (NIBS) have gathered substantial interest. NIBS interventions rely on brain plasticity mechanisms, with which neural pathways and circuits can be modified as a function of both internal (bodily) and external inputs, such as in response to environmental or controlled experimental stimuli, as in the case of NIBS interventions [21]. This phenomenon is present throughout the life span [21, 22], and it mimics the mechanisms of long-term potentiation/depression in glutamatergic synapses, leading to the NIBS-induced changes to outlast the period of stimulation [23]. Since altered excitability and plasticity are a hallmark in many neurological pathologies in modulating the relationship between brain insults and clinical outcome [24–26], great interest has been directed toward the possibility of leveraging on brain plasticity mechanisms to generate enduring modulations of activity in anatomical systems impacted by the disease or in spared neural networks interconnected with the former [27].

The most common form of NIBS is transcranial magnetic stimulation (TMS), consisting of a brief discharge of an electric current through a coil, inducing a focal magnetic field, that penetrates the scalp and the skull and secondarily generates an electric current, in accordance with the Faraday's principle of electromagnetic induction [28]. Depending on the frequency of stimulation with repetitive TMS (rTMS), either excitation (≥ 5 Hz: high-frequency rTMS) or inhibition (≤ 1 Hz: low-frequency rTMS) of the underlying neuronal activity of the stimulated area can be induced [29, 30]. Although rTMS is widely used, current clinical guidelines state a level A evidence (definite efficacy) only for the management of few pathologies, such as neuropathic pain, depression and hand motor recovery in the post-acute stages of stroke [31]. Evidence is less strong, yet promising, for the use of TMS in other pathologies, such as obsessive compulsive disorder [32]. On the other hand, the use of rTMS in AD is still debated due to considerable heterogeneity across studies and protocols, which relates back to the lack of (1) standardized stimulation parameters (intensity, frequency, duration of intervention), (2) knowledge of the optimal stimulation site and (3) the recruitment of large, well-characterized cohorts with a biomarker-confirmed diagnosis [33]. Due to these limitations, the FDA has not yet granted clearance for the commercial use of TMS devices in the treatment of AD pathology, as the amount of evidence collected so far is still not sufficient to clearly state its effectiveness at the clinical level [34].

To try to address some of these concerns, in the present study we conducted a meta-analysis of studies published in the last 10 years (2010–2021) aimed at disentangling the many protocols’ characteristics that might have acted as modulating factors for the success of rTMS interventional outcomes in AD patients. To our knowledge, no study before 2010 has applied rTMS in AD patients with protocols relevant to our research question. Differently from prior recent published work [35–39], we were interested in investigating whether protocols that considered participants-specific neuroimaging scans for the selection of *individualized* stimulation targets held more successful outcomes compared to those relying on a *generalized* targeting selection criteria. Our initial hypothesis was that the personalization of the stimulation site at the participant level should ensure greater protocol efficacy, compared to interventions relying on a “one fits all” paradigm, whereby the same stimulation site, generally chosen based on gross anatomical landmarks (e.g. the “5 cm rule” [40] for the identification of the Dorsolateral Prefrontal Cortex—DLPFC), is targeted across patients regardless of their interindividual anatomical differences. Indeed, the wild intersubject variability in the structural and functional organization of the brain calls for the development of personalized stimulation approaches. This has become a debated topic in the emerging field of precision medicine, which aims at estimating quantitative models of brain functioning—and its alterations—through the combination of the individual biochemical, functional, metabolic, morphological and neuropsychological profile [41]. Despite encouraging results on the personalization of rTMS interventions in the treatment of depression [42–44], individualized patient care is a desirable, still unmet, need in AD [45]. Indeed, our literature search has highlighted the presence of few published article where the stimulation target was personalized based on the individual anatomy, but there is still a substantial lack of trials using more articulated patients’ data, such as functional or tractography neuroimaging data. As a result, the level of personalization of the stimulation site might still have been suboptimal. Despite so, to our knowledge this is the first study trying to retrospectively investigate whether even a small degree of personalization could result in greater therapeutic responses compared to general targeting protocols.

On a second set of analyses, we compared additional protocol characteristics (i.e. different number of stimulation sites, pulse frequency, number of stimuli delivered, number of treatment sessions, concomitant cognitive training during stimulation) and participants’ characteristics (e.g. educational level) to identify other factors that might modulate the success of high-frequency rTMS protocols in AD. Despite the fact that other neurostimulation interventions are routinely applied as an attempt to ameliorate AD symptomatology (e.g. transcranial electrical stimulation), TMS shows the highest potential effectiveness [46]. For this reason, we decided to only review studies employing this methodology.

## Methods

### Search strategy and study selection

This study was designed in accordance with the Preferred Reporting Items for Systematic Reviews and Meta-Analyses (PRISMA) guidelines [47]. A systematic literature search was performed from January 2010 to February 2021 in PubMed, PsycINFO and Scopus databases. The keywords used were: “TMS” or “Transcranial Magnetic Stimulation” and “AD” or “Alzheimer’s disease” and their combination. The focus was on original, randomized, double-blind clinical trials designed for therapeutic purposes, with either parallel or crossover designs. Review papers and the references cited in the identified studies were used to extend the search for further relevant literature. Only studies written in English were considered.

The following inclusion criteria were used to identify eligible studies: (1) AD diagnosis based on well-defined diagnostic criteria, such as the Diagnostic and Statistical Manual of Mental Disorders (DSM) or the National Institute of Neurological and Communicative Disorders and Stroke/Alzheimer Disease and Related Disorders Association (NINCDS/ADRDA) criteria; (2) Mild to Moderate AD as determined based on NINCDS-ADRDA criteria for probable AD or a Mini Mental State Examination (MMSE) score within the range of mild (21–26) or moderate (10–20) AD and/or a Clinical Dementia Rating scale (CDR) score of 1 or 2 [48]. If present, diagnosis severity based on laboratory results, such as computed tomography, magnetic resonance imaging, positron emission tomography or lumbar puncture, was also considered. To even-out study comparison, additional inclusion criteria were: (3) cognitive performance scores assessed via global cognitive scales—e.g. the MMSE and/or the Alzheimer Disease Assessment Scale cognitive subscale (ADAS-cog)—at both baseline (pre-treatment) and immediately post-treatment assessments; (4) the use of high-frequency (≥ 5 Hz) rTMS protocols.

On the other hand, exclusion criteria included the following: (1) single-arm studies or studies without sham condition, to ensure control over placebo effects; (2) case reports, to ensure greater generalizability of the findings; (3) mixed-sample studies (severe AD or MCI patients)—unless patients’ data were reported separately for each severity group—since prior studies have shown greater effect of stimulation in milder patients [39, 49]; (4) AD patients with other concomitant forms of dementia (e.g. vascular dementia) or other comorbidities (e.g. depression) to ensure homogeneity across samples, as well as to limit unwanted confounding factors; (5) 1 Hz rTMS, that is, inhibitory stimulation protocols, to further reduce the heterogeneity across studies, especially since they represent the minority of the interventions in AD studies; (6) theta-burst stimulation protocols, since their effects remain debated in the literature [50, 51]; and (7) absence of behavioural data relating to immediate post-treatment global cognitive scales, since our aim was to address immediate cognitive effects of stimulation. The detailed list of studies excluded from this meta-analysis and their reasons is available in Table S1 of the Supplementary Materials.

**Fig. 1:**
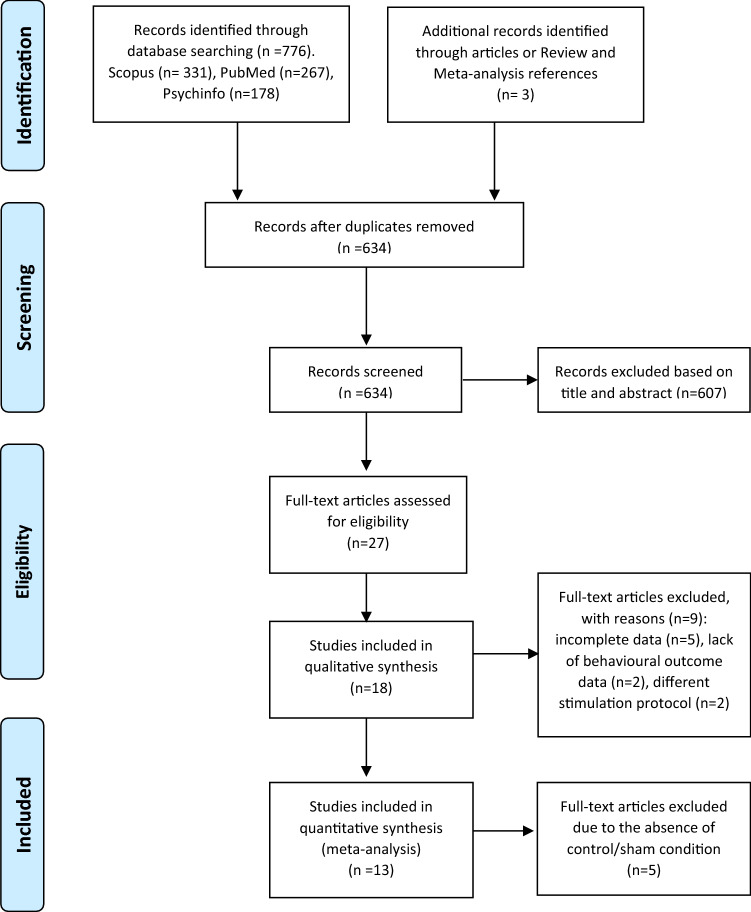
Diagram flow. Search strategy and study selection for the present meta-analytical study according to the PRISMA guidelines

In this study, we chose to focus on high-frequency rTMS interventions in mild-to-moderate AD patients based on prior literature evidence suggesting higher improvement rate in these patients [31, 39, 52, 53]. The study selection process is briefly summarized in Fig. 1.

### Data extraction and statistical analysis

A total of 13 articles [54–66] were included in our meta-analysis, for an overall sample of 427 AD participants. A summary of the demographic and clinical information of each study is reported in Table 1. Details on the intervention parameters and efficacy of treatment are reported in Table 2.

**Table 1:** Demographic and Clinical Information

Study information		Sample size		Demographical information						Clinical information			
Authors	Study design	Experimental condition		Gender ratio (%Female)		Age		Education		Active AD Medications	Diagnostic criteria	Disease duration/stage	
		Active	Sham	Active	Sham	Active	Sham	Active	Sham			Active	Sham
Ahmed et al. [54]	Parallel	15	15	66.6%	66.6%	65.9 ± 5.9	68.3 ± 4.9	Literate ≤ 6 years 26.7% > 6 years 20% Illiterate 53.3%	Literate ≤ 6 years 26.7% > 6 years 20% Illiterate 53.3%	None for at least 2 weeks before	NINCDS-ADRDA	3.9 ± 2.3/ mild-mod	4.4 ± 2.5/ mild-mod
Bagattini et al. [55]	Parallel	12	12	41.67%	41.67%	73.56 ± 4.91	73.35 ± 1.09	7.5 ± 3.75	7.33 ± 2.42	AChEI	Neurological examination	1.94 ± 0.7/ mild-mod	1.67 ± 1.3/ mild-mod
Brem et al. [56]	Parallel	16	8	75%	37.5%	69.25 ± 6.80	67.50 ± 10.27	14.25 ± 4.64	17.50 ± 4.00	AChEI and/or Memantine	DSM-V NIA-AA	NA/ mild-mod	NA/ mild-mod
Cotelli et al. [57]	Parallel	5	5	NA	NA	71.2 ± 6.1	74.4 ± 3.8	6.4 ± 1.3	4.8 ± 0.4	AChEI	NINCDS-ADRDA	NA/ moderate	NA/ moderate
Koch et al. [58]	Crossover	14		50%		70.0 ± 5.1		7.2 ± 3.0		NA	CSF and MRI	1.15 ± 0.43/ mild	
Lee et al. [59]	Parallel	18	9	55.6%	62.5%	72.1 ± 7.6	70.3 ± 4.8	9.9 ± 4.8	9.9 ± 3.7	AChEI	DSM-IV	NA/ mild-mod	NA/ mild-mod
Rabey et al. [60]	Parallel	7	8	71.43%	62.5%	72.6 ± 8.9	75.4 ± 9.07	NA	NA	AChEI and/or Memantine	DSM-IV	NA/ mild-mod	NA/ mild-mod
Rutherford et al. [61]	Crossover	10		60%		57–87		NA	NA	Stable dose for at least 3 months prior	Neurological examination	NA/ mild-mod	NA/ mild-mod
Sabbagh et al. [62]	Parallel	59	50	48,1%	42%	76.9	76.7	8th grade 5.1% High school 43% College 50.6% Other 1.3%	8th grade 8% High school 28% College 64% Other 0%	AChEI and/or Memantine	DSM-IV	1.7/ mild-mod	1.8/ mild-mod
Wu et al. [63]	Parallel	26	26	62%	58%	71.4 ± 4.8	71.9 ± 4.8	11.4 ± 2.7	11.5 ± 2.1	AChEI	NINCDS-ADRDA	5.1 ± 1.5/ moderate	5.1 ± 1.5/ moderate
Xingxing et al. [64]	Parallel	37	38	35.95%	36.84%	65.97 ± 8.47	64.58 ± 7.88	5.65 ± 3.21	6.75 ± 4.51	AChEI	DSM-V	3.7 ± 1.75/ mild-mod	3.97 ± 1.62/ mild-mod
Zhang et al. [65]	Parallel	15	13	80%	77%	69.00 ± 8.19	68.54 ± 7.93	12.40 ± 2.06	11.85 ± 2.38	Memantine	NINCDS-ADRDA + MRI	NA/ mild-mod	NA/ mild-mod
Zhao et al. [66]	Parallel	17	13	58.8%	54.8%	69.3 ± 5.8	71.4 ± 5.2	4.8 ± 1.9	4.9 ± 3.5	AChEI	DSM-IV	NA/ mild-mod	NA/ mild-mod

A summary of the main AD sample characteristics is reported for each study, including mean age, gender ratio, years of education, ongoing medical treatments at the time of stimulation and years of disease duration when available

*AChEI* acetylcholinesterase inhibitor, *DSM* Diagnostic and Statistical Manual of Mental Disorders, *NINCDS-ADRDA* National Institute of Neurological and Communicative Disorders and Stroke/Alzheimer disease and Related Disorders Association, *CSF* cerebrospinal fluid (lumbar puncture), *MRI* magnetic resonance imaging, *NA* not available/not reported information

**Table 2:** rTMS intervention details

Study information		TMS parameters					General cognition			Results
Authors	Study design	Frequency	Intensity	Stimulation site	Target	Pulses	Screening test	Mean ± SD		Main findings
								Baseline	After rTMS	
Ahmed et al. [54]	rTMS	20 Hz	90% RMT	R-DLPFC; L-DLPFC	GEN	2000pulses × 5 sessions	MMSE	18.4 ± 2.7	21.4 ± 3.2	Five daily sessions of 20 Hz rTMS on l-DLPFC and r-DLPFC lead to overall cognitive improvement in patients with mild to moderate AD, which was maintained for 3 months
Bagattini et al. [55]	rTMS + CT	20 Hz	100% RMT	L-DLPFC	GEN	2000pulses × 20 sessions	MMSE	21.62 ± 2.61	22.45 ± 1.89	The improvement in trained associative memory induced with rTMS is superior to CT alone, with less compromised and more educated patients showing greater benefit
Brem et al. [56]	rTMS + CT	10 Hz	120% RMT	L/R-IFG, L/R-STG, L/R-DLPFC	IND	1300pulses × 30 sessions	ADAS-Cog	ΔM = − 2.18 *S*_diff_ = 3.7		Combinatory treatment of rTMS and CT leads to significant cognitive improvement. Baseline TMS-induced plasticity is predictive of post-intervention changes in cognition
Cotelli et al. [57]	rTMS	20 Hz	100% RMT	L-DLPFC	GEN	2000pulses × 20 sessions	MMSE	16.2 ± 2.7	16.0 ± 3.3	Beneficial effects of rTMS on sentence comprehension in AD patients. No significant differences are found for other cognitive domains
Koch et al. [58]	rTMS	20 Hz	100% RMT	PRECUNEUS	IND	1600pulses × 30 sessions	MMSE	26.9 ± 1.9	27.3 ± 1.6	rTMS induces a selective improvement in episodic memory, but not in other cognitive domains. This improvement is accompanied by modulation of brain connectivity
Lee et al. [59]	rTMS + CT	10 Hz	90–110% RMT	Broca, Wernicke L/R- DLPFC, L/R-pSAC	IND	1200pulses × 30 sessions	ADAS-Cog	23.61 ± 6.40	19.33 ± 8.30	rTMS combined with CT represents a useful adjuvant therapy with cholinesterase inhibitors, particularly during the mild stage of AD and concerning memory and language domains
Rabey et al. [60]	rTMS + CT	10 Hz	90–110% RMT	Broca, Wernicke L/R- DLPFC,L/R-pSAC	IND	1300pulses × 54 sessions	ADAS-Cog	Δ*M* = − 3.76 *S*_diff_= 3.49		rTMS combined with CT provides a significant improvement in the mean ADAS-cog score and this improvement is greater than that achievable by using CT or TMS treatment alone
Rutherford et al. (2015)	rTMS	20 Hz	90–100% RMT	L/R-DLPFC	GEN	2000pulses × 13 sessions	ADAS-Cog	Δ*M* = − 4.00 *S*_diff_= 3.95		General cognitive skills improve, but the effect may not last longer than a week or two. A significant effect is seen in the MoCA scores, but not as much in the ADAS-cog scores
Sabbagh et al. [61]	rTMS + CT	10 Hz	110% RMT	Broca, Wernicke L/R- DLPFC, L/R-pSAC	IND	1300pulses × 30 sessions	ADAS-Cog	Δ*M* = − 0.706 *S*_diff_= 3.642		No statistically significant difference on ADAS-Cog between active and sham groups is found at the end of treatment. At follow up (5 months) only the active group shows a sustained improvement
Wu et al. [63]	rTMS	20 Hz	80% RMT	L-DLPFC	GEN	1200pulses × 20 sessions	ADAS-Cog	30.08 ± 6.07	24.16 ± 5.21	Combining drugs with rTMS adjunct treatment significantly improves both cognitive functioning and the behavioural and psychological symptoms of AD
Xingxing et al. [64]	rTMS	20 Hz	100% RMT	L-DLPFC	GEN	2000pulses × 30 sessions	ADAS-Cog	29.12 ± 5.97	26.23 ± 6.01	Small but significant improvement after rTMS treatment is reported compared with sham. Cortical LTP-like plasticity could predict treatment responses of cognitive improvements in AD
Zhang et al. [65]	rTMS + CT	10 Hz	90% RMT	L-DLPFC; L-LTL	GEN	1000pulses × 20 sessions	MMSE	ΔM = 3.27 *S*_diff_= 2.169		rTMS combined with CT improves cognitive function and ameliorates agitation and apathy in patients with mild and moderate AD by increasing the NAA/Cr metabolites ratio in the L-DLPFC
Zhao et al. [66]	rTMS	20 Hz	90% RMT	Parietal P3/P4 Posterior-temporal T5/T6	GEN	1000pulses × 20 sessions	ADAS-Cog	22.6 ± 5.9	18.5 ± 5.4	Patients in the active group significantly improve from baseline after 6 weeks of intervention. Improvement in episodic memory and language was higher in the mild AD group, compared to the moderate AD group

A summary of the main rTMS parameters used in each study is reported, including cognitive scores at the global cognitive scales before and immediately after the intervention. A brief summary of the main findings is also provided for each study

*CT* cognitive training, *RMT* resting motor threshold, *L* left, *R* right, *DLPFC* dorsolateral prefrontal cortex, *IFG* inferior frontal gyrus, *STG* superior temporal gyrus, *pSAC* parietal Somatosensory Association Cortices, *LTL* lateral temporal lobe, *GEN* generalized targeting, *IND* individualized targeting, *MMSE* Mini-Mental State Examination, *ADAS-Cog* Alzheimer’s Disease Assessment Scale (Cognitive Subscale), *ΔM* mean difference, *S*_*diff*_ pooled standard deviation

The effectiveness of rTMS in AD patients was investigated by comparing the changes in global cognition at the administered cognitive scales (MMSE or ADAS-cog) between the active and sham groups/conditions. For each study considered in the analyses, we extracted the mean difference between the post- and pre-treatment global cognition scores for both the sham and active groups/conditions (*M*_1_ and *M*_2_, respectively) as a measure of the rTMS-dependent change in global cognition. The corresponding standard deviations (SD) were also extracted when available; otherwise, they were calculated from the reported standard error of the mean (SE) and sample size (*n*) according to the formula: SD = SE * √*n*. In the case of longitudinal studies with multiple time-points, the immediate post-treatment scores were selected and compared to pre-treatment baseline performance scores. In studies where both MMSE and ADAS-cog scores were reported, measures from the latter were preferred. Prior literature evidence has indeed compared the sensitivity across different global cognitive scales and highlighted more precise measuring by means of the ADAS-cog scale [67]. Unfortunately, it would have been advisable to use more sensitive cognitive scales to better understand the effect of rTMS on the specific cognitive domains, both as a function of disease stage and site of stimulation. Indeed, in the view of personalized approaches, the use of appropriate, sensitive and precise cognitive evaluations is at least as important as the accurate dosing of stimulation parameters. However, the majority of studies we analyzed did not report extensive cognitive evaluations, making it hard to compare studies on specific domains, but only at the global cognitive level.

The Meta-Essentials workbooks were used for the meta-analysis [68]. For each study considered in the analyses, the t-statistic for the comparison of the rTMS-dependent change in global cognition between the active and sham groups/conditions was used to derive the corresponding effect size (Hedge’s *g*) and its confidence interval. For the studies reporting the F-statistic, its square root was taken to compute the t-statistic. If the statistic for the active vs. sham comparison of the rTMS-dependent change in global cognition was not reported in the original study, the authors were kindly asked via email to provide it; alternatively, an approximation of the t-statistic was computed from the available data in the article, including the sample size, mean difference and standard deviation of the rTMS-dependent changes in global cognition in the active and sham groups/conditions. The following formula was used for parallel trials:

$$t= \frac{{M}_{2}-{M}_{1}}{{\mathrm{SD}}_{\mathrm{pooled}}*\sqrt{\frac{1}{{n}_{1}}+\frac{1}{{n}_{2}}}},$$

where *M*_1_ and *M*_2_ refer to the mean rTMS-dependent changes in global cognition of the sham and active group, respectively, n_1_ and n_2_ refer to their sample sizes, and SD_pooled_ refer to their pooled SD, computed based on the corresponding SD_1_ and SD_2_ according to the formula.

$${\mathrm{SD}}_{\mathrm{pooled}}=\sqrt{\frac{\left({n}_{1}-1\right){{\mathrm{SD}}_{1}}^{2}+\left({n}_{2}-1\right){{\mathrm{SD}}_{2}}^{2}}{{n}_{1}+{n}_{2}-2}}.$$

For crossover trials, the t-statistic was computed as the standardized mean difference (*M*_diff_) of the mean rTMS-dependent changes in global cognition in the sham and active conditions according to the formula:

$$t=\sqrt{n}\frac{{M}_{\mathrm{diff}}}{{\mathrm{SD}}_{\mathrm{diff}}},$$

where SD_diff_ is the standard deviation of the differences of the mean rTMS-dependent changes in global cognition in the sham and active conditions.

The Cohen’s *d* effect size was then computed for each study according to the formula

$$d=t\sqrt{\frac{1}{{n}_{1}}+\frac{1}{{n}_{2}},}$$

for parallel trials and the formula

$$d=\frac{t}{\sqrt{n}},$$

for crossover trials.

Finally, the Hedges’ *g* effect size was computed as:

$$g=d\left(1-\frac{3}{4\left({n}_{1}+{n}_{2}\right)-9}\right).$$

Heterogeneity across studies was estimated based on the Cochrane’s *Q*, *I*^2^, and *T*^2^, which reflect, respectively, the variability of the effects around the weighted average effect, the proportion of observed variance reflecting real differences in effect size and an estimate of the variance of the true effect size [69]. Random effects model with 95% confidence interval was run to summarize the effect size, as well as to test for differences between studies’ groups in our subgroup analyses (see next paragraphs).

Finally, to represent the risk of publication bias, funnel plots were created in Meta-Essentials [68] and the resultant effect sizes adjusted by means of the trim-and-fill procedure [70].

### Moderator analysis

A moderator analysis (meta-regression) was conducted to test the effect that the total number of stimulation pulses had on the effect size of the considered studies. This predictor was obtained by multiplying the number of pulses per session by the number of total sessions in the study protocol.

### Subgroup analyses

In our main hypothesis, we speculated that interventions where the stimulation site is *individualized* based on the participant’s neuroimaging data would result in greater stimulation effects compared to interventions relying on a *generalized* target selection procedure, where general anatomical landmarks are used to approximately identify the same stimulation site across participants (e.g. studies relying on the electroencephalography (EEG) 10–20 electrode cap disposition or the “5 cm rule” to determine the position of the DLPFC). Indeed, recent efforts in the direction of personalized interventions in the treatment of depression have been proven successful [42–44]. However, to our knowledge, no such investigation has yet being carried out in the field of AD research. Given the purpose of this study, additional subgroup comparisons were then carried out in order to understand the role that several other variables, related to both the experimental protocol and the sample characteristics, might have had in modulating the effectiveness of the treatment, as suggested by a recent meta-analysis [39]. In particular, we compared the efficacy of studies stimulating: (i) ≤ 10 Hz versus > 10 Hz frequency; (ii) a single region (DLPFC) versus multiple brain regions stimulated sequentially; (iii) patients with low versus high education (> 8 years of education); (iv) the presence of concomitant cognitive training or not.

Analysis of variance (ANOVA) models were then run to test for significant differences in the effect sizes between groups of studies.

### Quality assessment

The methodological quality of 11, out of 13, parallel, double-blind, randomized clinical trials included in the meta-analyses was evaluated by two authors independently (LD, AM) with the Cochrane Risk of Bias tool (RoB2) for randomized trials [71]. The risk of bias scale covers five domains of bias: randomisation process (D1), deviations from the intended intervention (D2), missing outcome data (D3), measurement of the outcome (D4), selection of the reported results (D5). The remaining assessment of the two crossover, double-blind, randomized trials was carried out by the same two authors (LD, AM) by means of the RoB2 tool for crossover trials. In addition to the above-mentioned domains, this scale also investigates the bias arising from period and carryover effects (DS). In case of conflicting judgments, a third author’s (EA) opinion was asked.

## Results

### Efficacy of high-frequency stimulation in AD

**Fig. 2:**
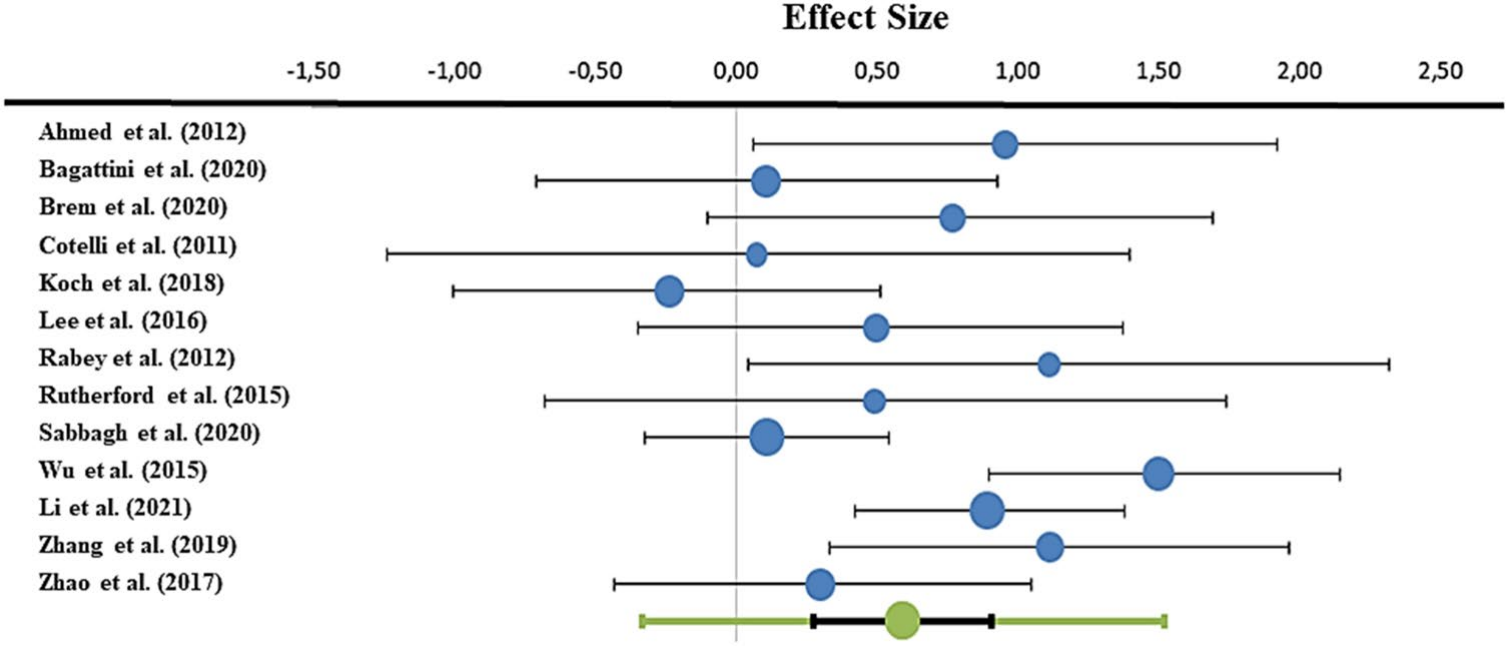
Forest plot. Effect sizes of individual studies and of their pooled effect (bottom row) are reported. Meta-analytic evidence suggests a favourable effect of rTMS in the amelioration of global cognitive functioning in mild–moderate AD patients. Dots’ size represents the relative weight of each study

The pooled results of the 13 studies included in our study indicated that rTMS could significantly improve participants’ global cognitive functioning as evidenced by an increase in the MMSE scores, or a decrease in the ADAS-Cog scores (Hedges' *g* = 0.59, SD = 0.15; CI = 0.27–0.91, *p* < 0.001) in the random effects model analysis, showing a moderate heterogeneity (*p*_Q_ = 0.009; *I*^2^ = 54.88%, *T*^2^ = 0.16). Figure 2 reports the effect sizes of the single studies, as well as the combined effect size, in form of a forest plot.

### Subgroups analyses

**Fig. 3:**
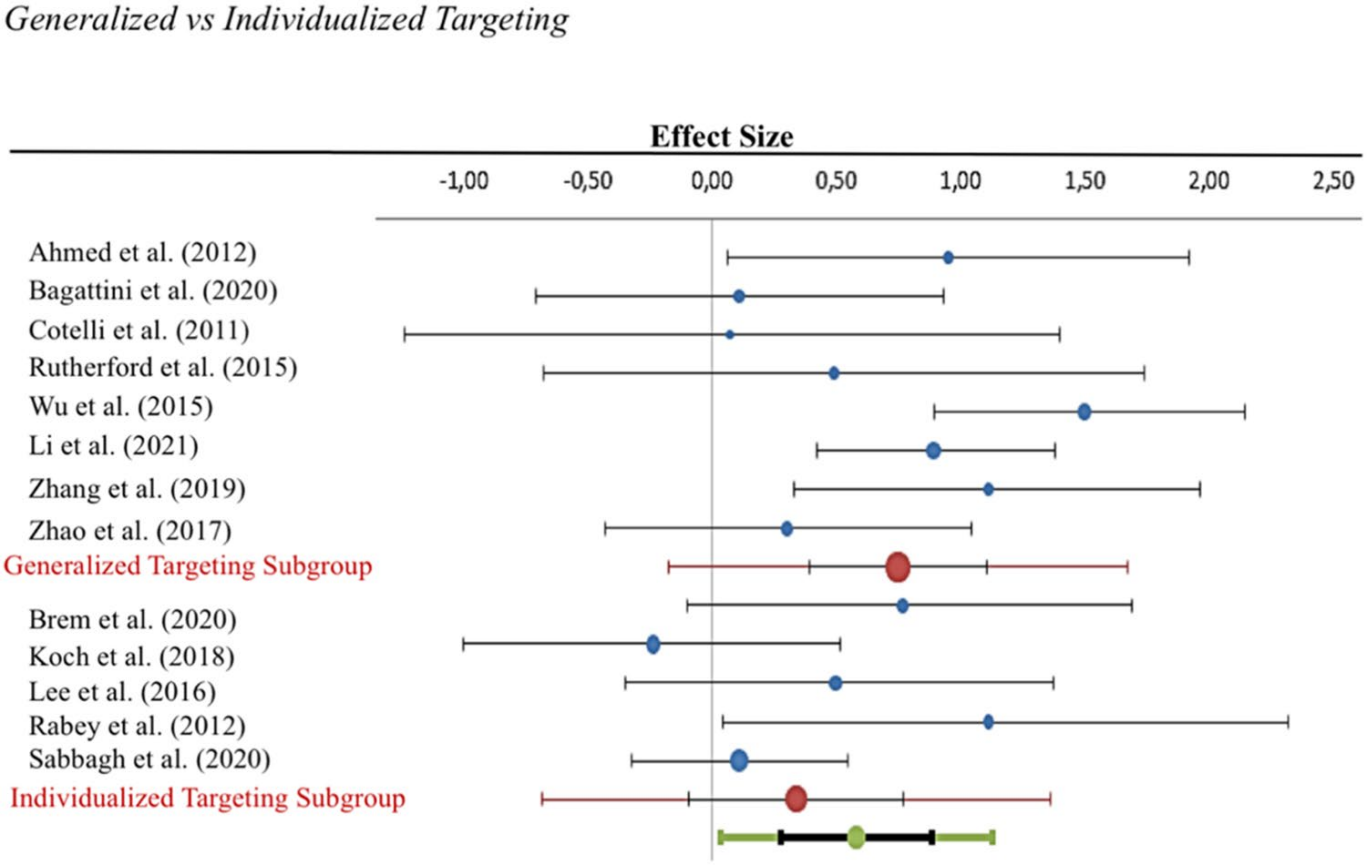
Forest plots of *individualized* vs *generalized* target selection subgroup analyses. No significant differences could be observed in the effect sizes of studies as a function of target selection procedure

In contrast to our main hypothesis, the subgroup analysis comparing the efficacy of studies employing *individualized* (*n* = 5, Hedges’ *g* = 0.34; CI = -0.10–0.77, *I*^2^ = 39.36%, *p*_Q=_0.159, *T*^2^ = 0.09) versus *generalized* (*n* = 8, Hedges’ *g* = 0.75; CI = 0.39–1.11, *I*^2^ = 45,72%, *p*_Q=_ 0.075, *T*^2^ = 0.12) targeting (Fig. 3) did not show a significant difference (*p* = 0.146).

**Fig. 4:**
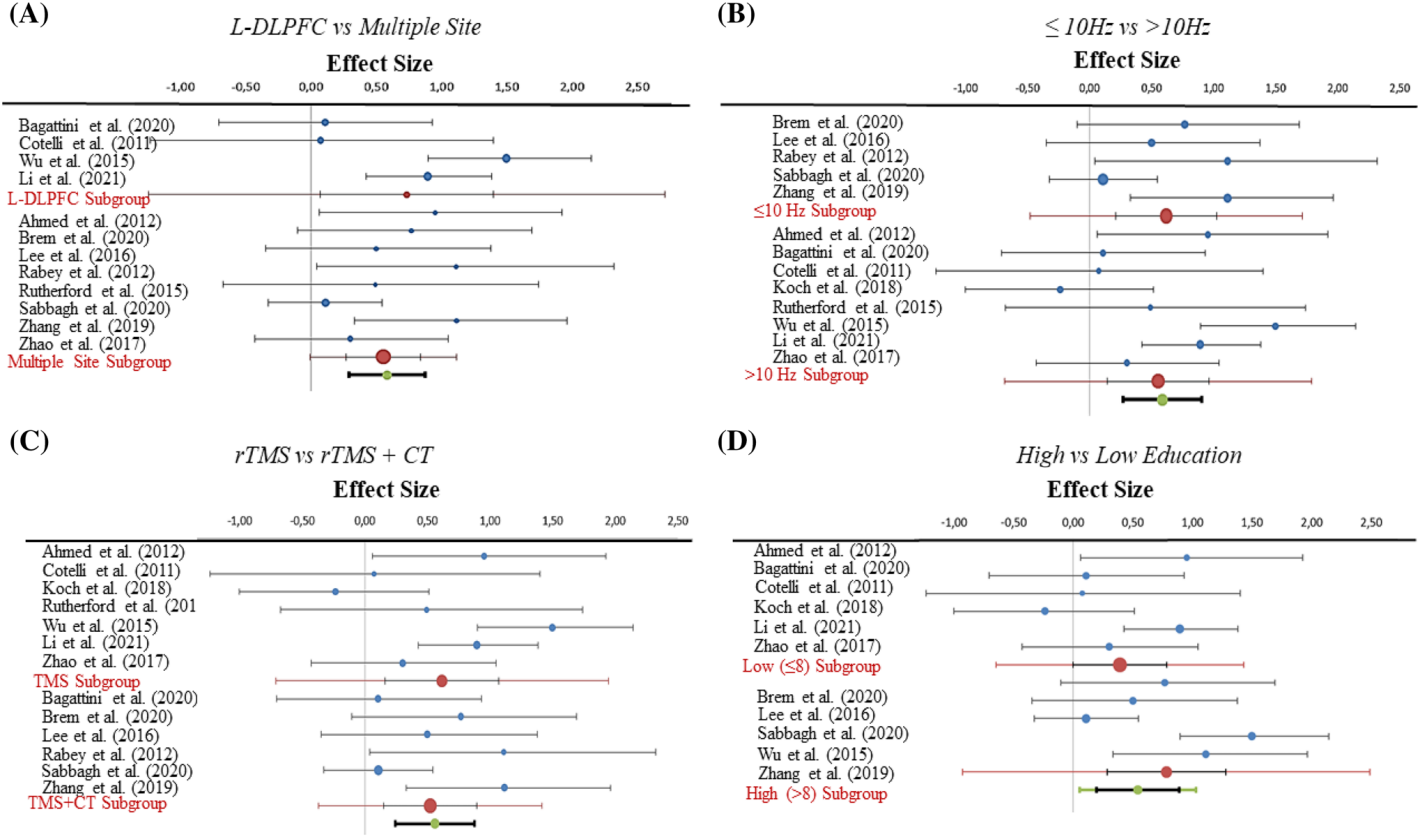
Forest plots of subgroup analyses. No significant differences could be observed in the effect sizes of studies as a function of: number of stimulation sites (L-DLPFC vs multiple sites) (**A**), frequency of stimulation (≤ 10 Hz vs > 10 Hz) (**B**), TMS only or TMS combined with cognitive training (CT) (**C**), high (> 8) vs low (≤ 8) patients’ education level (**D**)

Additional analyses aimed at understanding which stimulation factors might have an impact on rTMS efficacy in AD also did not show any statistically significant difference. In particular, at the protocol level, stimulation of the left DLPFC (*n* = 4, Hedges’ *g* = 0.73; CI = − 0.07–1.40, *I*^2^ = 69.33%, *p*_Q=_0.021, *T*^2^ = 0.27) versus stimulation of multiple sites (*n* = 8, Hedges’ *g* = 0.55; CI = − 0.27–0.84, i^2^ = 18.99%, *p*_Q=_0.279, i^2^ = 0.03) did not impact the results (*p* = 0.614) (Fig. 4, panel A). Furthermore, the efficacy of studies where the stimulation frequency was ≤ 10 Hz (*n* = 5, Hedges’ *g* = 0.62; CI = − 0.21–1.02, *I*^2^ = 45.11%, *p*_Q=_0.121, *T*^2^ = 0.11), compared to those with stimulation frequency > 10 Hz (*n* = 8, Hedges’ *g* = 0.55; CI = − 0.14–0.97, *I*^2^ = 62.55%, *p*_Q=_0.009, *T*^2^ = 0.23) did not show any significant difference (*p* = 0.837) (Fig. 4, panel B). At the sample level, studies in which participants received both cognitive training and stimulation (*n* = 6, Hedges’ *g* = 0.52; CI = 0.15–0.89, *I*^2^ = 38.12%, *p*_Q=_0.152, *T*^2^ = 0.09), compared to those receiving stimulation alone (*n* = 7, Hedges’ *g* = 0.62; CI = 0.16–1.07, *I*^2^ = 63.92%, *p*_Q=_0.011, *T*^2^ = 0.24), did not show any significant difference (*p* = 0.764) (Fig. 4, panel C). Similarly, no difference in the efficacy of stimulation was observed between studies in which participants had a high (*n* = 5, Hedges’ g = 0.78; CI = 0.28–1.28, *I*^2^ = 73.58%, *p*_Q=_0.004, *T*^2^ = 0.31) and low (*n* = 6, Hedges’ *g* = 0.39; CI = − 0.00–0.79, *I*^2^ = 47.46%, *p*_Q=_0.090, *T*^2^ = 0.12) level of education (*p* = 0.287) (Fig. 4, panel D).

### Moderator analysis

**Fig. 5:**
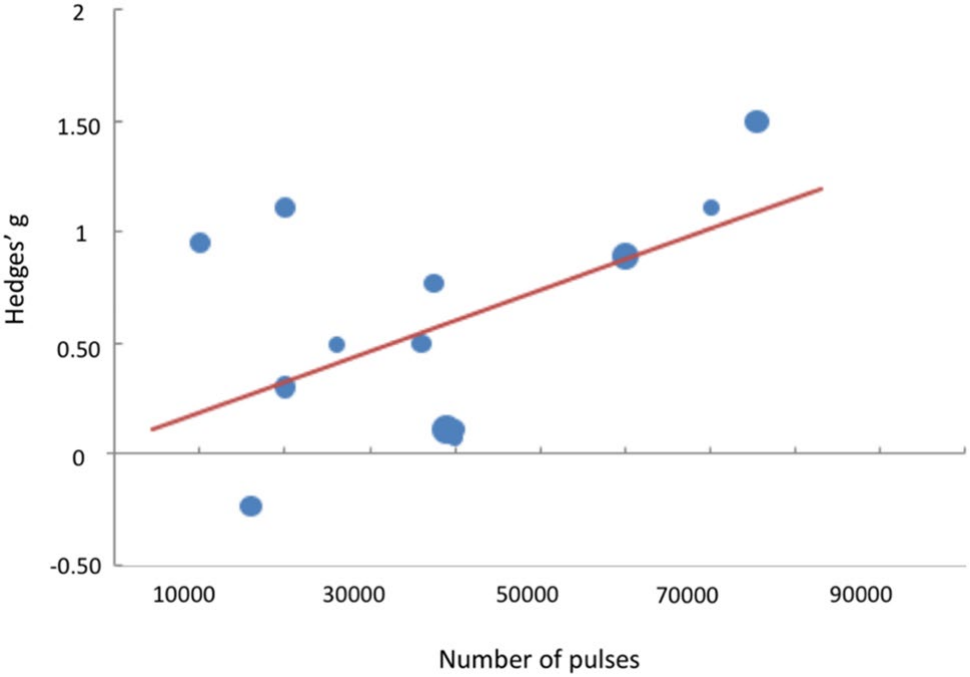
Moderator analysis. Linear regression analyses revealed a significant correlation between the total number of pulses per protocol and the studies’ effect size. Dots’ size is indicative of each study relative weight

The moderator analysis revealed a significant correlation between the total number of pulses delivered per protocol and the study effect size (*ß* = 0.54, *p* = 0.038, *R*^2^ = 29.16%) (Fig. 5), with the following regression equation: *g* = 0.0474 + Pulses*1.38*10^–5^, where *Pulses* indicates the total number of pulses delivered. Based on this analysis, approximately 39,000 pulses are needed to have an effect size of *g* = 0.59, equivalent to the pooled effect size we found.

### Risk of bias analyses

**Fig. 6:**
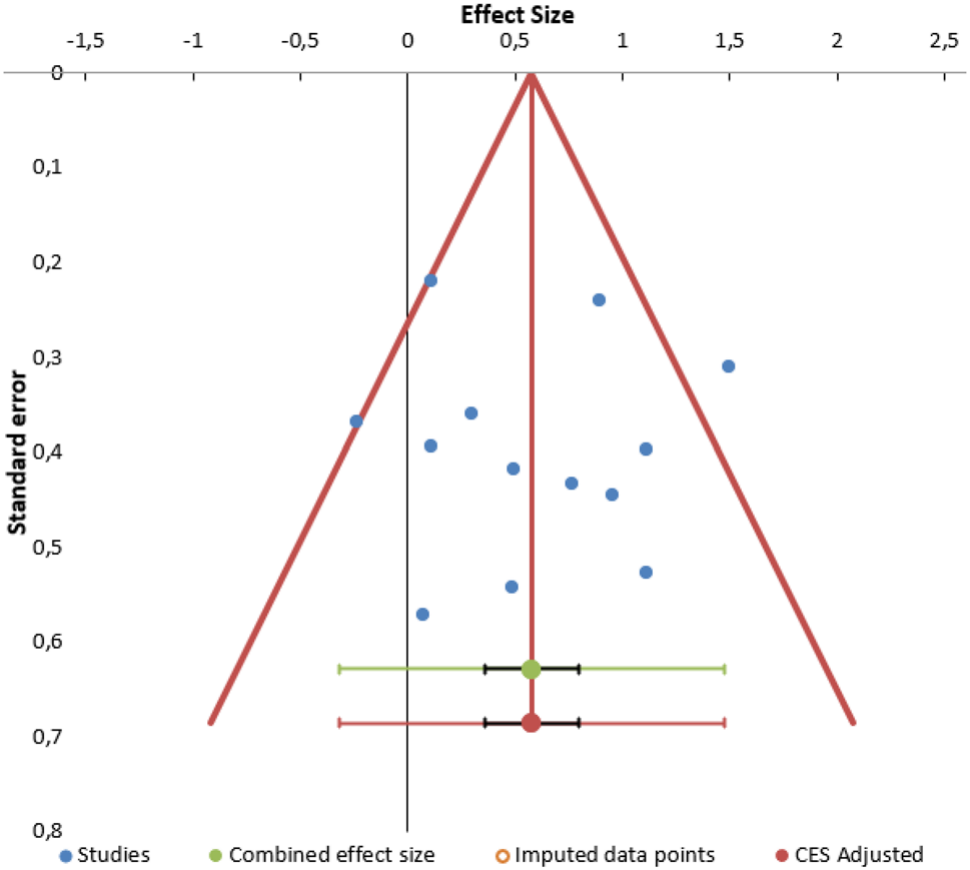
Funnel plot for Publication Bias analysis. Standard errors and effect sizes of the included studies are shown in the funnel plot. No significant publication bias was detected

As shown in Fig. 6, the funnel plot is visually symmetrical at the Begg & Mazumdar’s test (*p* = 0.807), as well as the Egger’s test (*p* = 0.981), thus suggesting that no significant publication bias was present in the selected studies. As a result, no fill-and-trim procedure was carried out to adjust the effect sizes.

In addition, each study was evaluated on the risk of bias across 5 domains, proving an overall low to moderate risk (Table 3).

**Table 3:**
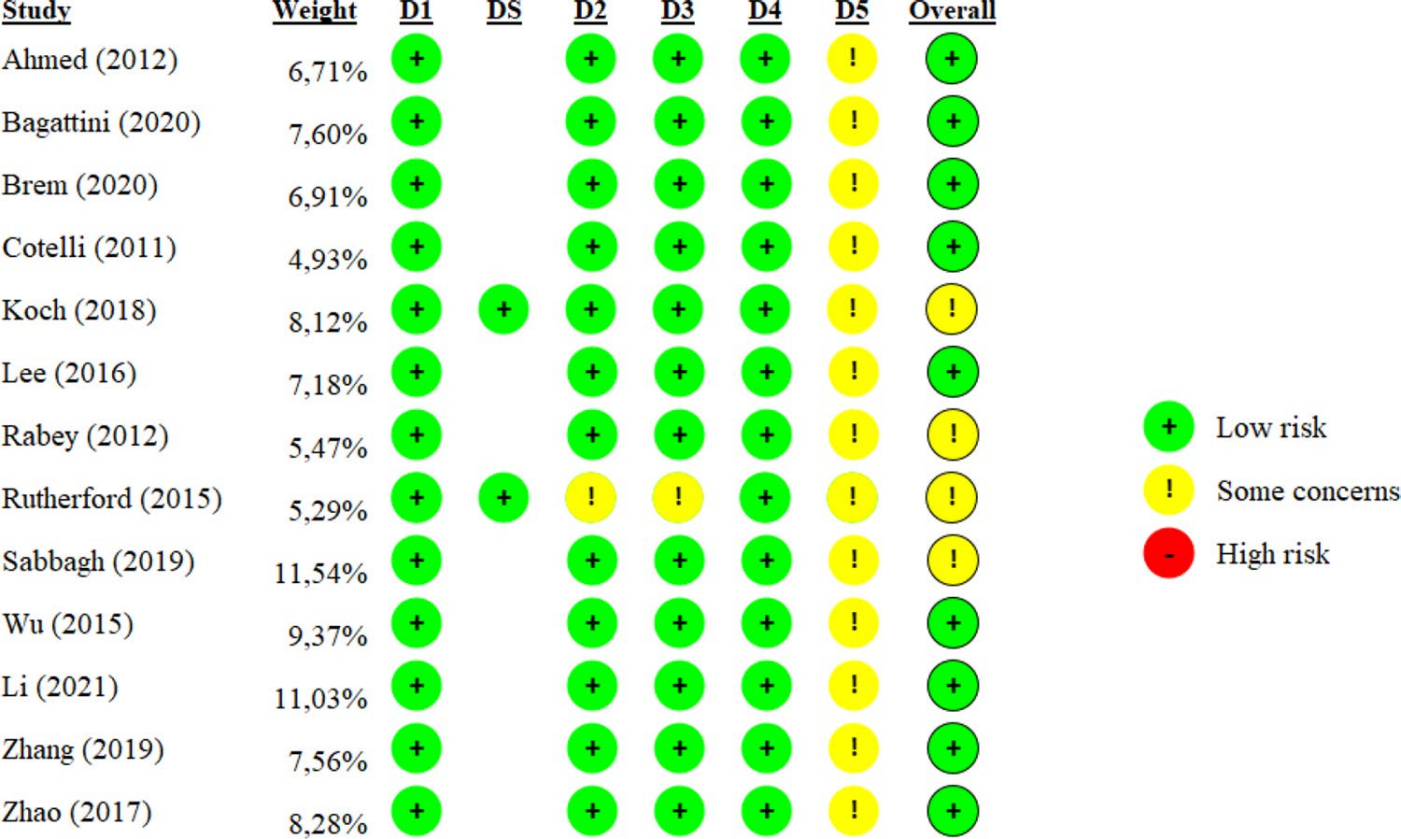
Risk of bias

Each study was evaluated based on the RoB2 tool for the assessment of the risk of bias across 5 domains: randomisation process (D1), deviations from the intended intervention (D2), missing outcome data (D3), measurement of the outcome (D4), selection of the reported result (D5). In addition, for the 2 studies with a crossover design, the bias arising from period and carryover effects (DS) was also assessed. The overall column refers to the overall quality of the study, considering the average across the single domains

## Discussion

Repetitive TMS is a safe and promising noninvasive intervention for a variety of neuropsychiatric conditions [72, 73]. However, the most recent evidence-based guidelines on the therapeutic use of rTMS have shown debating results for its use in the management of the AD pathology, reaching only a level C evidence (“possibly effective or ineffective”) [31]. Several factors might have led to this conclusion, especially since the high heterogeneity in the administered protocols makes it hard to compare across studies [33].

The main aim of our study was that of addressing which protocols’ characteristics are more likely to explain the heterogeneity in the results across studies. To do so, we started by investigating the importance that personalized stimulation target selection might have in AD studies employing rTMS to boost or preserve residual cognitive functioning. The rationale for personalized care is not new in the neuroscience field and rather stands from the need to develop in-person care via the use of therapies that are biomarker-guided and grounded on the biological characteristics of the individual patient [74–76]. In the clinical management of the AD pathology, progress in this direction has been made with the establishment of the Alzheimer Precision Medicine Initiative (APMI), which aims at estimating quantitative models of the disease through the combination of the individual biochemical, functional, metabolic, morphological and neuropsychological profile [41]. As for what concerns the personalization of rTMS interventions, this has also been of interest in recent literature studies. For example, in the year 2020 alone, several applications of rTMS in the management of depression have reported benefits following individualized target selection. Interestingly, personalization of the stimulation site has been achieved through a variety of approaches, including (i) the identification of the target site based on a localization of circuits and the corresponding scalp targets that relate to post-TMS improvement in depressive symptoms—the so-called symptom-response map—[44]; (ii) the targeting of functionally derived individual brain parcels [43]; or (iii) based on the individual connectivity profile of small regions of interest in the treated pathology, such as the subgenual cingulate cortex [42]. Given the high relevance of such recent discoveries and the interest for the same principles to be applied in the management of the AD pathology [45], we were interested in determining whether similar favourable results could be observed by retrospectively looking at the effect size of studies employing individual data for the selection of the stimulation site. In this regard, our study confirmed prior literature evidence on the efficacy of rTMS interventions in AD patients [33, 35, 37, 38, 49, 52, 77, 78], but we were not able to demonstrate that personalized interventions held better outcomes than those employing more general targeting approaches. One possible explanation is that studies individualizing the stimulation site relied on patients’ anatomical magnetic resonance imaging, but not on their functional or structural connectivity profile. This might have led to a smaller degree of individualization, not capable of detecting neural re-arrangements typical of the pathological aging brain. Indeed, although individual brains vary substantially in their cytoarchitectonic and macrostructural anatomy, they also do so in their functional organization with respect to the structural anatomy [79–81]. In particular, functional connectivity profiles are characterized by stable individual features, with modest variations from task-state and day-to-day variability [82], as well as great individual specificity [83], making them desirable features to be accounted for when aiming at the individualization of stimulation sites. In principle, indeed, precise targeting of individual-specific functional brain networks should improve the efficacy of NIBS interventions [84]. Similarly, recent evidence has shown that the structural connectivity profile is a good predictor of the propagation of the TMS signal [85], with possible implications in its use as a variable of interest in stimulation target definition. We might hence argue that past effort in the individualization of stimulation sites has not been of sufficient extent. Future interventions will need to move beyond the use of simple anatomical data and rather consider functional and structural connectivity profiles for a better characterization of the individual brain organization and of its targeting.

Nevertheless, even when other variables related to the experimental protocol and the sample characteristics were compared (i.e. differences in stimulation frequency, targeting of single or multiple stimulation sites, the education level or the additive effect of concurrent cognitive training), no significant differences were detected across studies employing different combinations of these factors. Of interest, prior work had instead highlighted meaningful differences in this direction, showing significantly greater cognitive improvement in participants with a high level of education, who received rTMS treatment at multiple stimulation sites, for more than 10 sessions, at 20 Hz instead of lower frequencies and that possibly received simultaneous cognitive training [39]. However, the different statistical models used to compare effect sizes in our work compared to prior published work, may explain the conflicting results. In fact, in the work of Wang and colleagues, when the heterogeneity value *I*^2^ was < 50%, a fixed effects model was applied, in contrast to our choice to always apply a random effects model. This methodological choice was made on the basis of a couple of reasons. First, there were insufficient good reasons to believe that all studies were functionally identical [86]: given the widespread clinical and/or methodological differences found in the included studies, statistical heterogeneity was inevitable [87]. Second, being the aim of a meta-analysis that of including independent research studies, the hypothesis of a common effect size was not tenable [88]. Indeed, a fixed effects model requires the assumption of a common effect size, which can lead to the lack of generalizability outside the well-defined population included in the analysis [86]. Furthermore, the use of a fixed effects model in the presence of heterogeneity could lead to an underestimation of the variability of the treatment and a consequent deviation from the true conclusions of the study [89]. In light of this, a random approach analysis seemed to be more appropriate in order to quantify the heterogeneity of the effects across studies, to be able to incorporate this variation in the confidence intervals of the data distribution, to test the adequacy of the models that attempt to explain this variation, and overall to obtain accurate effect size estimates for each study [90].

On the other hand, we were able to replicate the positive association between the total number of pulses delivered per protocol and studies’ effect size in our moderator analysis. Indeed, the periodic repetition of rTMS stimulation, usually administered on a daily basis (within 24 h from the previous session), can lead to cumulative plastic changes that can generate long-lasting neuromodulatory effects [30], on top of the 30–60 min after-effects observed following a single stimulation [91]. However, it is important to consider that the total number of pulses was treated as a collective measure (number of pulses per session by the number of total sessions in the study protocol), and that we did not consider the sparseness of pulses distribution throughout the protocol. In this sense, past studies have highlighted more favourable cognitive outcomes in patients receiving at least 10 stimulation sessions [39]. Only recently, systematic studies have been carried out to determine the impact of different number of pulses in remission rates from major depressive disorder, with inconclusive results [92]. In regard of the AD pathology, it would be of interest for future studies to better characterize such dosing parameters, in terms of the relationship between pulses and sessions.

## Limitations

Based on our present findings, several considerations need to be made. First of all, our analyses might have suffered from a somehow limited amount of placebo-controlled trials and the often limited number of study participants. Indeed, our initial literature search identified 18 studies in which AD patients received rTMS treatment; of those, only 13 were included in the analyses as 5 of them lacked the presence of a control group (see Fig. 1). Secondly, we observed that the included studies in this meta-analyses had relatively small sample sizes, as demonstrated by the fact that 10 out of the 13 studies considered have a sample size of less than, or equal to, 30 participants (including 3 studies with less than 20 participants), which might have also undermined the possibility of adequate and in-depth statistical analysis.

Finally, as already stressed in the past, there is substantial heterogeneity across studies in terms of the rTMS parameters, sites of stimulation and samples’ characteristics [33], making it hard to conduct clear-cut subgroup analyses. Beyond this, there are several other criticalities related to rTMS interventions in a broader sense. In particular, several intrinsic factors to the stimulation can result in inter-subject variability, contributing to the heterogeneity of the results. Some of these factors include: (1) participants’ age and gender, (2) the distance between the scalp and the cerebral cortex in modulating the amount of current that reaches the brain tissue, (3) the richness and integrity of the white matter tracts underlying the stimulation site and finally (4) genetic phenotypes [30, 93]. In this regard, it has been shown that the modulatory effect of TMS is reduced in participants carrying the “Val66Met” allele of the brain-derived neurotrophic factor gene (BDNF) [94]. When applied to the AD population, these limitations are further worsened by other factors, such as the lack of well-defined diagnostic criteria, as AD patients are mostly identified based on probable diagnoses in the absence of appropriate disease biomarkers (e.g. positron emission tomography-derived amyloidosis and tau maps [95], lumbar puncture) and the even greater scalp-to-cortex distance due to the widespread cortical atrophy [96]. Furthermore, we observed significant heterogeneity in the neuropsychological batteries employed to measure patients’ cognitive functioning. The majority of the studies relies on global cognitive scales (such as the MMSE and the ADAS-Cog) which, although useful, might lack sufficient sensitivity to adequately monitor patients’ improvement over time. Although some of the included studies reported information related to single cognitive domains, the lack of coherent assessment protocols makes it hard to conduct a meta-analysis on the effect of rTMS in specific cognitive functions, such as episodic memory abilities or visuo-spatial orientation, as those are among the first functions affected by the disease [6].

Despite the aforementioned limits, few precautionary measures can be taken in trying to overcome them. For example, to control for the induced electrical currents in patients with diffuse atrophic patterns, multi-scale computational approaches can be used to model the induced TMS activation in the underlying neural substrates [97]. The use of head models allows to represent the type of activated neural elements, the spatial extent of such activations, and how spatial and temporal parameters of TMS determine threshold and site of activation, particularly when considering the complicated and subject-dependent human brain geometry [98, 99]. Indeed, various studies have investigated the inter-subject variability of the TMS-induced electric field and have shown consensus that both the strength of the electric field and the location of the hot spot depend on individual anatomical differences [100, 101].

Secondly, future studies may make use of a deeper understanding of the individual network topology to guide stimulation interventions. One example is represented by studies directed at targeting individual-specific “hub” brain areas, based on the assumption that modulation of highly connected regions should have greater impact on cognition than nonhub brain areas. Indeed, regions that connect to several other networks, known as connector hubs, are believed to be crucial for information transfer and between-network communication within the brain [102]. In line with this assumption, a recent study reported that the inhibition of a hub via its TMS stimulation interrupted information processing during working memory tasks with a substantial difference with respect to when a nonhub site was targeted instead, despite both targets being separated by only few centimetres along the right middle frontal gyrus [103]. As stated by the authors, such findings further stress the notion that individual-specific network features are functionally relevant and could be used in principle as stimulation sites in future TMS interventions. Indeed, the use of network-guided TMS has long been suggested in the literature, based on the notion that different network alteration profiles can be appreciated across neurological disorders [78, 104]. Based on this rationale, several studies have highlighted the possibility to act on those alterations in trying to restore healthy brain network patterns [45, 105], whereby brain networks are employed for both the definition of the target and for the monitoring of the efficacy of the stimulation treatment [106].

Finally, numerous studies have assessed the relationship between the ongoing oscillatory activity of the brain (as measured via EEG) and the physiological responses to TMS. General TMS devices do not adjust the output stimulation based on the real-time brain activity information, despite recent evidence that the ongoing oscillatory activity of the brain, especially its phase, may affect stimulation effects [107, 108]. The automatic electronic adjustment of the stimulation based on previous responses, also known as closed-loop stimulation, is widely employed in other neuromodulation approaches, such as transcranial alternating current stimulation (tACS), which specifically aims at the entrainment of the underlying brain physiological activity. The main rationale stands from the notion that neurons are more likely to fire in correspondence to a specific time point in their spiking cycle, such as that the closer the stimulation is delivered to that narrow time window, the greater the likelihood of synaptic strengthening or weakening [109]. However, current results remain controversial, as many studies have reported offline effects that are independent from oscillatory entrainment mechanisms and rather reflect more general changes in plasticity, thus not specific to the stimulation been tuned to the underlying neural firing [109]. Still, it would be desirable for future studies to try to adapt similar closed-loop procedures for the fine tuning of the frequency at which rTMS is delivered based on the ongoing underlying brain activity. Indeed, recent evidence suggests that stimulation based on real-time knowledge of the state of activity of the brain (for example represented by the sinusoidal oscillation of a specific frequency band) can help control the efficacy of the induced plasticity changes and induce more specific neuromodulatory effects [110, 111].

## Conclusion

This study shows that rTMS is a promising intervention in the treatment of patients with mild to moderate AD and highlights the positive correlation between effect size reported in the reviewed studies and the total number of pulses administered during the intervention. Further investigations will be necessary to better clarify which combinations of protocol characteristics and parameters are most efficient in promoting residual cognitive functioning in AD patients. Of outermost importance is the development of standardized approaches to reduce inter-study heterogeneity and foster reliable findings. Future rTMS protocols in the AD population would benefit from: (1) an in-depth and biomarker-guided diagnostic framework, (2) stimulation target selection that takes into account individual differences in the underlying anatomical, structural/functional connectivity and oscillatory activity patterns and finally (3) the consistent use of neuropsychological test batteries for the comparable measurement and monitoring of patients’ cognitive functioning across studies. Finally, it would be desirable to better assess the long-term efficacy of repeated rTMS interventions, as follow-up timing is also highly variable between studies.

Given the complex and multifactorial nature of the AD pathology, multidisciplinary efforts are needed in order to integrate inter-individual variability as part of the foundation of the intervention strategy, rather than examining it strictly post hoc as a mere confounding variable. Practical examples include the development of personalized stimulation protocols through a multiscale approach and based on the individual clinical make-up. In this sense, models could be constructed integrating knowledge on the cellular to large-scale networks alterations (including functional and structural connectome organization) of the individual patients to personalize stimulation in at least three of its parameters: site selection, intensity and frequency of stimulation. This would greatly help ensuring that the most critical region is reached by electrical currents strong enough to modulate remaining synaptic plasticity mechanisms.

## Supplementary Information

Below is the link to the electronic supplementary material.

Supplementary file1 (DOCX 18 kb)
