# Supplementary material for: Transcranial magnetic stimulation treatment in Alzheimer’s disease: a meta-analysis of its efficacy as a function of protocol characteristics and degree of personalization
Source: J Neurol. 2022 Jul 4;269(10):5283–301. doi: 10.1007/s00415-022-11236-2 (PMC9468063; doi:10.1007/s00415-022-11236-2)
Supplement: Supplementary file 1 — Supplementary file1 (DOCX 18 kb) [file 415_2022_11236_MOESM1_ESM.docx]

**Transcranial Magnetic Stimulation treatment in Alzheimer’s Disease: a meta-analysis of its efficacy as a function of protocol characteristics and degree of personalization**

Arianna Menardi^1,2,†*^, Lisa Dotti^3†^, Ettore Ambrosini^1,2,3^, Antonino Vallesi^1,2^

1. Department of Neuroscience, University of Padova, Padova, Italy
2. Padova Neuroscience Center, University of Padova, Padova, Italy
3. Department of General Psychology, University of Padova, Padova, Italy

**Running Head:** TMS efficacy in AD

**Number of Figures:** 6

**Number of Tables:** 3

**Abstract words count:** 221

† Contributed Equally

***Corresponding author:**

Arianna Menardi

Department of Neuroscience, University of Padova

Padova, Italy, 35121

arianna.menardi@gmail.com

**Table S1. Comprehensive list of studies excluded from the meta-analysis and their reasons.**

| **Study** | **Reasons for rejection** |
| --- | --- |
| **Alcalá-Lozano**, R., Morelos-Santana, E., Cortés-Sotres, J. F., Garza-Villarreal, E. A., Sosa-Ortiz, A. L., & González-Olvera, J. J. (2018). Similar clinical improvement and maintenance after rTMS at 5 Hz using a simple vs. Complex protocol in Alzheimer’s disease.Brain Stimulation: Basic, Translational, and Clinical Research in Neuromodulation | **Incomplete data.** Lack of sham/control group |
| **Anderkova**, L., Eliasova, I., Marecek, R., Janousova, E., & Rektorova, I. (2015). Distinct Pattern of Gray Matter Atrophy in Mild Alzheimer’s Disease Impacts on Cognitive Outcomes of Noninvasive Brain Stimulation*.* Journal of Alzheimer’s Disease | **Lack of behavioural outcome data**.  The effect of the stimulation was measured using three tests: Trail Making Test (TMT), Stroop test (ST), and Complex Visual Scene Encoding Task (CVSET). Screening tests (including MMSE) were administered only at baseline. |
| **Bentwich**, J., Dobronevsky, E., Aichenbaum, S., Shorer, R., Peretz, R., Khaigrekht, M., … Rabey, J. M. (2011). Beneficial effect of repetitive transcranial magnetic stimulation combined with cognitive training for the treatment of Alzheimer’s disease: A proof of concept study. Journal of Neural Transmission | **Incomplete data.** Lack of sham/control group |
| **Devi**, G., Voss, H. U., Levine, D., Abrassart, D., Heier, L., Halper, J., … Lowe, S. (2014). Open-Label, Short-Term, Repetitive Transcranial Magnetic Stimulation in Patients With Alzheimer’s Disease With Functional Imaging Correlates and Literature Review. American Journal of Alzheimer’s Disease & Other Dementias. | **Incomplete data.** Lack of sham/control group |
| **Eliasova**, I., Anderkova, L., Marecek, R., & Rektorova, I. (2014). Non-invasive brain stimulation of the right inferior frontal gyrus may improve attention in early Alzheimer’s disease: A pilot study. Journal of the Neurological Sciences | **Lack of behavioural outcome data.**  Trail Making Test (TMT), Stroop test, and CVSET test were considered for the measurement of post-treatment behavioural outcome. Screening tests (including MMSE) were administered only at baseline. |
| **Nguyen**, J.-P., Suarez, A., Saout, E. L., Meignier, M., Nizard, J., & Lefaucheur, J.-P. (2018). Combining cognitive training and multi-site rTMS to improve cognitive functions in Alzheimer’s disease. Brain Stimulation: Basic, Translational, and Clinical Research in Neuromodulation | **Incomplete data.** Lack of sham/control group |
| **Turriziani**, P., Smirni, D., Mangano, G. R., Zappalà, G., Giustiniani, A., Cipolotti, L., & Oliveri, M. (2019). Low-Frequency Repetitive Transcranial Magnetic Stimulation of the Right Dorsolateral Prefrontal Cortex Enhances Recognition Memory in Alzheimer’s Disease. Journal of Alzheimer’s Disease | **Different stimulation protocol.**  Inhibitory rTMS stimulation. |
| **Rabey**, J. M., & Dobronevsky, E. (2016). Repetitive transcranial magnetic stimulation (rTMS) combined with cognitive training is a safe and effective modality for the treatment of Alzheimer’s disease: Clinical experience*.* Journal of Neural Transmission | **Incomplete data.** Lack of sham/control group |
| **Wu**, X., Ji, G.-J., Geng, Z., Zhou, S., Yan, Y., Wei, L., … Wang, K. (2020). Strengthened theta-burst transcranial magnetic stimulation as an adjunctive treatment for Alzheimer’s disease: An open-label pilot study. Brain Stimulation: Basic, Translational, and Clinical Research in Neuromodulation | **Different stimulation protocol.** iTBS protocol |
